# Interplay of anxiety, depression, vascular function, and biomarkers in post-myocardial infarction patients

**DOI:** 10.3389/fphys.2025.1594889

**Published:** 2025-05-26

**Authors:** Jan Kafol, Borut Jug, Mojca Božič Mijovski, Jure Tršan, Daniel Košuta, Marko Novaković

**Affiliations:** ^1^ University Medical Centre Ljubljana, Department of Vascular Diseases, Ljubljana, Slovenia; ^2^ University of Ljubljana, Faculty of Medicine, Ljubljana, Slovenia; ^3^ University of Ljubljana, Faculty of Pharmacy, Ljubljana, Slovenia

**Keywords:** anxiety, depression, cardiac rehabilitation, coronary artery disease, myocardial infarction

## Abstract

**Background and aims:**

Coronary artery disease (CAD) is a leading cause of mortality. Depression and anxiety are common in CAD patients and negatively affect quality of life, physical functioning, and adherence to cardiac rehabilitation (CR) programs. This study aimed to identify possible associations with clinically relevant parameters, vascular function and blood biomarkers.

**Methods:**

Participants were consecutively recruited during cardiac rehabilitation intake visits at the University Medical Centre Ljubljana within 4 months of myocardial infarction (MI). Hospital Anxiety and Depression Scale (HADS) scores were analyzed in relation to endothelial function (assessed with flow-mediated dilation), arterial stiffness, and blood biomarkers (fibrinogen, endocan, and brain-derived neurotrophic factor [BDNF]) in post-MI patients. All vascular and biomarker assessments were performed within 5 days of questionnaire completion and prior to the start of rehabilitation.

**Results:**

There were 105 patients included in the study. The median age was 56 years (49–62), and 80.0% of participants were male. Clinically relevant anxiety and depression were present in 29.5% and 21.9% of participants, respectively. Anxiety was significantly associated with younger age, higher body mass index, and increased arterial stiffness, with total HADS scores negatively correlated with age. Endothelial function showed no significant associations with HADS scores. Vital signs showed no significant differences, except for slightly higher systolic blood pressure in those with clinically relevant depression. Fibrinogen levels were significantly higher in participants with anxiety and depression, while endocan and BDNF levels were lower in those with anxiety.

**Conclusion:**

Depression and especially anxiety are significantly associated with endothelial function and relevant biomarkers in post-MI patients. However, as HADS is a screening tool and not a diagnostic instrument, and given the study’s observational design, findings reflect associations rather than causality. Routine screening and targeted mental health support within CR programs might improve participation, enhance cardiovascular recovery, and optimize long-term outcomes. These findings underscore the clinical importance of psychological assessment in the early post-MI period and support the integration of mental health evaluation into cardiovascular care.

## Introduction

Coronary artery disease (CAD) remains a leading cause of mortality worldwide (Mensah et al., 2023).

Depression affects 20%–40% of cardiac patients, particularly those in middle and older age groups, and is strongly associated with CAD progression, diminished quality of life, impaired physical function, recurrent cardiac events, and increased mortality risk (Celano and Huffman, 2011). Anxiety disorders are even more prevalent, affecting up to 70% of individuals with CAD, with similar age distributions (Silarova et al., 2014). Both conditions further compromise physical function, reduce quality of life, and contribute to premature mortality (Chen et al., 2019). Moreover, anxiety and depression pose significant barriers to both attendance and successful completion of cardiac rehabilitation (CR) following myocardial infarction (MI). Studies indicate that 18% of CR participants exhibit depressive symptoms, while 28% experience anxiety. Patients with these conditions are significantly less likely to adhere to rehabilitation programs compared to those without such symptoms (Rao et al., 2020).

Given the severe burden of CAD, current guidelines advocate for screening and treatment of these symptoms (Visseren et al., 2021; Virani et al., 2023). Routine screening for depression in patients post-MI, followed by appropriate treatment, has been shown to improve long-term cardiac outcomes (Kim et al., 2021). One such screening tool is the Hospital Anxiety and Depression Scale (HADS), a reliable self-assessment instrument for detecting anxiety and depression in hospital outpatient settings, with strong performance in identifying symptoms and assessing severity across various patient groups (Bjelland et al., 2002; Zigmond and Snaith, 1983).

Given that both depression and anxiety are well-established risk factors for CAD, this study aimed to assess their association with key clinical parameters, vascular function, and blood biomarkers in patients within 4 months after MI and prior to the start of CR (Visseren et al., 2021; Virani et al., 2023). The objective was to identify which parameters are most affected by psychological distress and to determine how addressing anxiety and depression during rehabilitation might yield the greatest clinical benefit. This was a hypothesis-driven observational study. Our primary hypothesis was that clinically relevant anxiety and depression would be associated with adverse vascular parameters, particularly increased arterial stiffness (pulse wave velocity [PWV]) and impaired endothelial function (flow-mediated dilation [FMD]). The secondary hypothesis was that these psychological conditions would be linked to alterations in blood biomarkers related to cardiovascular risk, specifically elevated fibrinogen levels and reduced concentrations of endocan and brain-derived neurotrophic factor [BDNF]. We also hypothesized that anxiety and depression would be more prevalent in younger patients and those with higher body mass index [BMI].

## Methods

### Study design

We evaluated baseline HADS scores and their associations with key clinical parameters, including endothelial function, arterial stiffness, and blood biomarkers (Zigmond and Snaith, 1983), to explore the relationship between psychological factors and cardiovascular health before the start of CR.

The study was approved by the National Medical Ethics Committee and conducted in accordance with the Declaration of Helsinki. All participants provided written informed consent before enrollment.

### Study participants

**FIGURE 1:**
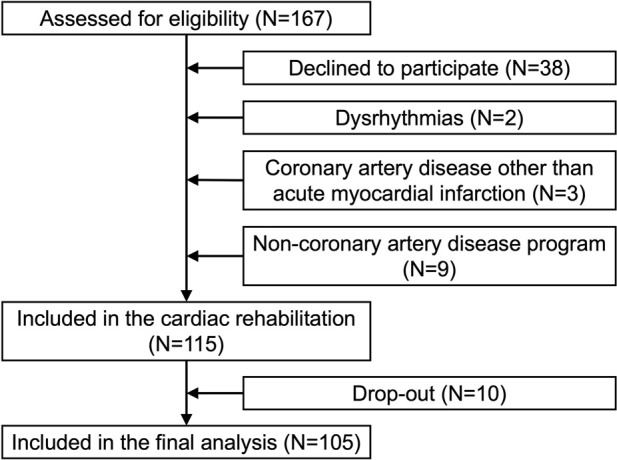
Prisma flow diagram of participant recruitment, exclusion, and inclusion in the study.

We recruited adult participants (aged 36–85 years, 80% male) at the Centre for Preventive Cardiology (see Figure 1), Department of Vascular Diseases, University Medical Centre Ljubljana, Slovenia. Participants were prospectively recruited during their intake evaluation at the rehabilitation center, where a cardiologist assessed their eligibility for cardiac rehabilitation following MI and simultaneously screened them for inclusion in the study. Eligible individuals had experienced a MI within the 4 months before completing the HADS questionnaire and underwent blood collection, endothelial function assessment, and arterial stiffness measurement within 5 days of completing the questionnaire. A total of 105 participants were included in the study. While a formal *a priori* sample size calculation was not conducted, the study aimed to detect moderate correlations (e.g., r = 0.3) with 80% power and a two-sided alpha of 0.05. This requires a minimum of 84 participants, indicating that our sample size was adequate for detecting such associations.

Exclusion criteria included acute illness (e.g., active infection, acute inflammation, or any new or worsening condition requiring urgent medical attention), recent (<1 month) non-cardiovascular conditions requiring hospitalization or emergency specialist care, unstable or poorly controlled dysrhythmias, pregnancy, and intellectual developmental disorders. Patients with previously diagnosed psychiatric disorders documented in their medical records were also excluded from the study.

### Study visit

Clinical data and routine blood samples were collected at least 3 days before the start of the CR program. Anthropometric measurements (body height, body weight) were measured by established methods. Arterial blood pressure and heart rate were measured in a sitting position after a few minutes of rest with an automated sphygmomanometer.

### Hospital Anxiety and Depression Scale

The HADS questionnaire was administered in written form, in Slovenian, the native language of all participants. Participants were given ample time to complete it independently, and completion of the HADS was a prerequisite for study inclusion. Clinically relevant anxiety or depression was defined as a score greater than 7 in the respective component of the HADS (Zigmond and Snaith, 1983). The HADS is a validated screening tool designed for use in nonpsychiatric clinical populations, with good internal consistency (Cronbach’s α = 0.83 for anxiety and 0.82 for depression), and optimal sensitivity and specificity of approximately 0.80 for both subscales at a cut-off score of ≥8 (Bjelland et al., 2002).

### Endothelial function

Endothelial function was assessed via FMD using the Aloka ProSound α7 ultrasound with a 10 MHz transducer, following established guidelines (Thijssen et al., 2019). To minimize diurnal variation, measurements were conducted in the morning. Participants were instructed to refrain from exercise, alcohol, smoking, caffeine, and antioxidant vitamins before testing, while a light breakfast was permitted to prevent discomfort.

Before measurements, participants rested supine in a quiet, air-conditioned room for 15 min, with the right brachial artery scanned longitudinally about 5 cm above the antecubital fossa. For consistency, the probe position was documented for each participant, and an insonation angle of <60°–70° was used. A baseline image was obtained, and a cuff was then inflated on the forearm to 50 mmHg above systolic pressure for 4.5 min. After deflation, a second image was taken 60 s later. The FMD was calculated as the percentage change in arterial diameter post-hyperemia relative to baseline.

### Arterial stiffness

Arterial stiffness was assessed through PWV, using a 10 MHz transducer on the Aloka ProSound α7 ultrasound with high-resolution echo tracking software. The procedure followed validated echo-tracking protocols and expert consensus recommendations (Laurent et al., 2006). PWV is a recognized surrogate marker of aortic stiffness and cardiovascular risk, with established reference values from large European population studies (Reference Values for Arterial Stiffness' Collaboration, 2010). Blood pressure was measured on the left upper arm, while arterial stiffness was measured on the right common carotid artery (RCCA). Participants were positioned supine with heads elevated at 45° and tilted left by 30°.

The RCCA was scanned about 2 cm before the carotid bifurcation, and the ultrasound cursor was placed on the anterior and posterior artery walls. Using systolic and diastolic pressures, the software analyzed waveforms from arterial wall displacement and automatically calculated the PWV over ten heartbeats. Calibration of blood pressure was repeated twice with six consecutive readings each time, and the reported PWV values were the average of 12 measurements.

Vascular assessments were conducted by two skilled observers, with additional intra- and inter-observer reliability testing performed. For FMD, intra- and inter-rater reliability coefficients were 0.906 and 0.821, respectively, and for PWV, 0.976 and 0.969, respectively.

### Blood biomarkers

From each participant blood sample was drawn from the antecubital vein into a 4.5 mL coagulation tube (Becton Dickinson, United States). Within 1 hour from collection platelet-poor plasma was prepared with 20-min centrifugation at 2,000 × g and 15°C. Half milliliter aliquots of plasma were frozen and stored at ≤−70°C until analysis. In thawed plasma fibrinogen levels were measured on an automated coagulation analyzer CS-2500 (Sysmex, Japan) with the Dade® Thrombin Reagent (Siemens Healthineers, Germany), while levels of endocan and BDNF were determined with xMAP^©^ technology using magnetic beads coupled with specific antibodies (all R&D Systems, United States) on a MagPix instrument (Luminex Corporation, United States).

### Statistical analysis

Data were collected and analyzed using Microsoft Office Excel 365 (Microsoft Corporation, United States), SPSS Statistics version 26.0 (IBM, United States), and R version 4.4.1 (R Foundation for Statistical Computing, Austria). Missing values were left as blanks and not substituted. Outliers were included in the statistical analysis. Numerical variables were assessed for normality using graphical methods and statistical tests (Shapiro-Wilk and Kolmogorov-Smirnov). As most numerical data were non-normally distributed, results are presented as medians with first to third quartile in the brackets (Q1–Q3). Levene’s test was used to assess the equality of variances. To compare variables between groups, the Mann-Whitney U test was used for non-normally distributed variables, and the independent samples t-test for normally distributed variables. For categorical data, the Chi-squared test was applied to 2 × 2 tables. The relationship between two variables was assessed using Spearman’s correlation, due to the non-normal distribution of at least one of the variables. All statistical tests were two-tailed, and a p-value ≤0.05 was considered statistically significant.

## Results

### Baseline characteristics

Among the 105 participants included in the analysis, all were of Caucasian origin. Detailed sociodemographic data and baseline characteristics, including age distribution and HADS scores, are summarized in Table 1.

**TABLE 1:** Sociodemographic and psychological characteristics of the study population (N = 105).

Characteristic	Value
Sex: Male	84 (80.0%)
Sex: Female	21 (20.0%)
Age	56 (49–62)
HADS total score	10 (6–15)
HADS Anxiety score	5 (3–8)
Clinically relevant anxiety†	31 (29.5%)
HADS Depression score	4 (3–7)
Clinically relevant depression†	23 (21.9%)

Data are presented as absolute frequency (proportion in %) and median (first quartile–third quartile).

Legend: HASD, hospital anxiety and depression scale.

Footnotes: † Clinically relevant anxiety or depression was defined as a score greater than 7 in the respective component of the HADS.

### Comparison of clinical parameters

**FIGURE 2:**
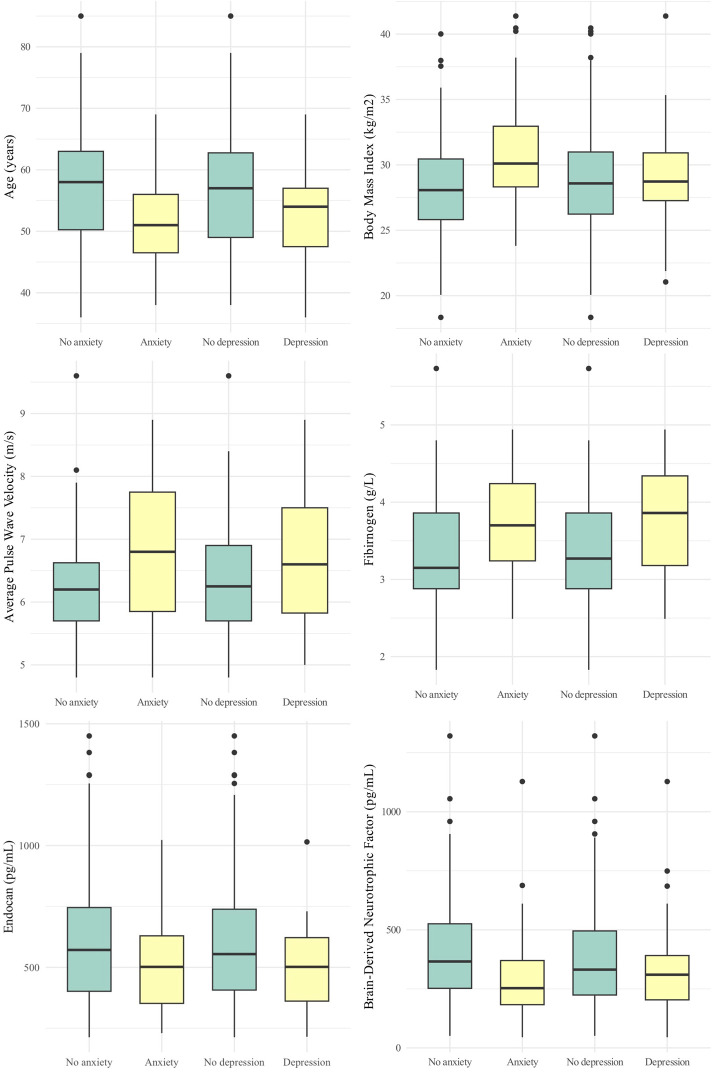
Comparison of key parameters by anxiety and depression status: Boxplots for age, body mass index, average pulse wave velocity, fibrinogen, endocan, and brain-derived neurotrophic factor.

**FIGURE 3:**
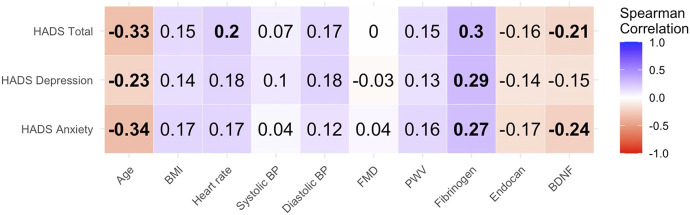
Heatmap of Spearman correlations between scores on the Hospital Anxiety and Depression Scale (HADS) — including anxiety, depression, and total scores—and selected clinical, vascular, and biomarker parameters in post-myocardial infarction patients (N = 105). Each cell represents the strength and direction of the correlation between a HADS domain and a clinical variable. Blue shades indicate positive correlations, red shades indicate negative correlations, and the color intensity reflects correlation strength. Bolded values denote statistically significant associations (p < 0.05). Correlations were calculated using Spearman’s rank method. Abbreviations: BMI = body mass index; BP = blood pressure; FMD = flow-mediated dilation; PWV = pulse wave velocity; BDNF = brain-derived neurotrophic factor.

Table 2 summarizes the comparisons of various clinical parameters by anxiety and depression status (see also Figure 2 for visual comparisons), while Figure 3 displays the correlations between HADS scores and selected clinical, vascular, and biomarker parameters.

**TABLE 2:** Comparison of various clinical parameters between individuals with and without anxiety and depression (N = 105).

Parameter	No anxiety (N = 74)	Anxiety (N = 31)	p-value	No depression (N = 82)	Depression (N = 23)	p-value
Males	61 (72.6%)	23 (27.4%)	0.336†	66 (78.6%)	18 (21.4%)	0.813†
Females	13 (61.9%)	8 (38.1%)		16 (76.2%)	5 (23.8%)	
Age (years)	58 (50–63)	51 (46–56)	0.006‡	57 (49–63)	54 (46–58)	0.077‡
BMI (kg/m^2^)	28.07 (25.79–30.52)	30.10 (28.04–33.46)	0.004§	28.58 (26.17–31.01)	28.73 (27.15–31.35)	0.792§
Heart rate (BPM)	69.00 (62.00–74.25)	71.00 (63.00–81.00)	0.132‡	68.50 (61.00–75.00)	71.00 (65.00–80.00)	0.138‡
Systolic BP (mmHg)	120.50 (111.00–131.25)	122.00 (115.00–141.00)	0.191§	120.50 (111.00–132.75)	129.00 (119.00–142.00)	0.047§
Diastolic BP (mmHg)	78.00 (70.00–82.25)	81.00 (73.00–88.00)	0.064§	78.00 (70.75–83.25)	80.00 (73.00–88.00)	0.135§
FMD (%)	4.18 (1.50–7.57)	4.50 (1.92–8.47)	0.553§	4.34 (1.64–7.37)	3.10 (1.08–9.38)	0.825§
PWV (m/s)^a^	6.20 (5.70–6.68)	6.80 (5.78–7.85)	0.027§	6.25 (5.70–6.90)	6.60 (5.80–7.60)	0.164§
Fibrinogen (g/L)^b^	3.15 (2.86–3.86)	3.70 (3.21–4.34)	0.020‡	3.27 (2.88–3.86)	3.86 (3.15–4.34)	0.047‡
Endocan (pg/mL)	571.50 (400.50–763.75)	502.00 (333.00–632.00)	0.047§	554.50 (403.25–742.50)	502.00 (352.00–643.00)	0.061§
BDNF (pg/mL)	366.00 (250.25–531.50)	253.00 (182.00–371.00)	0.016§	331.50 (219.00–502.25)	310.00 (184.00–405.00)	0.474§

Data are presented as absolute frequency (proportion in %) and median (first quartile–third quartile).

Legend: BMI, body mass index; BPM, beats per minute; BP, blood pressure; FMD, Flow-mediated dilation; PWV, pulse wave velocity; BDNF, Brain-derived neurotrophic factor.

Footnotes:^a^ N = 102;^b^ N = 104; † Pearson Chi-Square Test; ‡ Independent Samples t-test; § Mann-Whitney U Test.

No significant difference was observed in the prevalence of clinically relevant anxiety or depression between males and females (p = 0.336 and 0.813, respectively), nor was sex significantly associated with HADS scores. Although the median total HADS score was slightly higher in females (11.0 [8.0–14.5] vs. 9.5 [5.0–15.0]), the difference was not statistically significant (p = 0.191). Similarly, no significant difference was found for the anxiety section of HADS (7.0 [4.5–8.5] vs. 5.0 [3.0–8.0]; p = 0.075). Median depression scores were also comparable between females and males (4.0 [3.0–7.5] vs. 4.0 [2.0–7.0]; p = 0.609). However, participants with clinically relevant anxiety were significantly younger than those without (p = 0.006). Age was also significantly negatively correlated with the total HADS score (p = 0.001), as well as with scores for the anxiety (p < 0.001) and depression (p = 0.002).

There was a significant difference in BMI between those with and without clinically relevant anxiety (p = 0.004), while BMI was comparable between participants with or without clinically relevant depression. Vital signs, including heart rate, systolic, and diastolic blood pressure, showed no significant differences in relation to clinically relevant anxiety or depression, except for a slightly higher systolic blood pressure in participants with clinically relevant depression (p = 0.047). Likewise, no significant differences were found in FMD. However, heart rate showed a significant correlation with total HADS scores (p = 0.045).

Assessment of arterial stiffness via average PWV indicated significantly higher PWV in those with clinically relevant anxiety (p = 0.027), while clinically relevant depression was not associated with PWV (p = 0.164).

Among blood biomarkers, fibrinogen levels showed strong correlations with HADS score (total: p = 0.002; anxiety section: p = 0.006; depression section: p = 0.003), with significantly higher fibrinogen levels observed in individuals with both clinically relevant anxiety (p = 0.002) and clinically relevant depression (p = 0.047). Endocan and BDNF levels were notably lower in individuals with clinically relevant anxiety (p = 0.047 and p = 0.016, respectively), while differences in participants with and without clinically relevant depression were not statistically significant (p = 0.061 and p = 0.474, respectively). BDNF also displayed significant correlations with both total HADS (p = 0.028) and anxiety section scores (p = 0.014).

## Discussion

In this study, clinically relevant anxiety and depression were present in 29.5% and 21.9% of participants, respectively. Anxiety was more prevalent in younger individuals and associated with higher BMI and increased arterial stiffness (PWV). While most vital signs showed no significant differences, systolic blood pressure was slightly higher in those with depression. Elevated fibrinogen levels were observed in participants with both anxiety and depression, while endocan and BDNF levels were lower in those with anxiety. These findings underscore the physiological impact of psychological distress and highlight the importance of integrating mental health support into CR programs to optimize patient outcomes.

Screening for anxiety and depression in post-MI patients undergoing CR is crucial, not only because these conditions are significant risk factors for CAD, impacting mortality and other key outcomes, but also because they influence adherence to CR programs. Given that CR is essential for recovery and reintegration into daily life and work, addressing these psychological factors could improve participation and long-term cardiovascular health (Rao et al., 2020; Visseren et al., 2021; Virani et al., 2023).

Our study found no significant difference in rates of clinically relevant anxiety and depression between men and women, which contrasts with trends in the general population, where females are more than twice as likely as males to experience mood disorders—often attributed to hormonal differences (McHenry et al., 2014). Although our sample size was small, our findings suggest that chronic conditions like CAD may be a more impactful factor in driving anxiety and depression, effectively neutralizing the typical sex differences seen in the general population. Moreover, the shared experience of a major cardiovascular event, such as MI, may level emotional responses across genders. In this clinical context, both men and women may feel more comfortable expressing psychological distress, reducing gender-based reporting differences typically seen in community settings. However, it is important to acknowledge that 80% of our study participants were male. This sex imbalance reflects real-world clinical patterns, where significantly more men experience MI at a younger age—an age at which they are more likely to be referred for and capable of participating in cardiac rehabilitation. Women tend to develop MI at older ages, when functional limitations and comorbidities often preclude participation in structured rehabilitation programs (Schulte and Mayrovitz, 2023). As such, while our findings may be generalizable to younger male post-MI patients entering rehabilitation, caution is warranted when extending them to older or female populations. Future studies with larger and more sex-balanced cohorts are needed to confirm whether similar associations hold true across sexes.

Another key finding of our study was that patients with clinically relevant anxiety were significantly younger than those without, and both anxiety and depression showed a significant negative association with age. While older adults generally report lower prevalence rates of these conditions compared to younger and middle-aged adults, they remain particularly vulnerable due to unique life stressors and health challenges that heighten their susceptibility to anxiety and depression (Vink et al., 2008; Fiske et al., 2009; Villarroel and Terlizzi, 2020; Terlizzi and Villarroel, 2020). Additionally, experiencing a MI at a younger age can be a greater psychological shock, as it disrupts expectations of health, career, and personal life, potentially leading to heightened anxiety and emotional distress (Lavie and Milani, 2006; Wu et al., 2022).

Research shows that anxiety, depression, and psychosocial stress are linked to BMI, with obesity increasing the risk of mood and anxiety disorders, and vice versa (Eik-Nes et al., 2022; de Wit et al., 2022; Luppino et al., 2010). In our study, 88 participants (83.8%) had a BMI above 24.9 kg/m^2^ (overweight) and 39 (37.1%) had a BMI above 29.9 kg/m^2^ (obese). Participants with clinically relevant anxiety had significantly higher BMI compared to those without, while the difference in BMI between those with and without clinically relevant depression was not significant, likely due to the small sample size, though other factors may also play a role. Further research is needed to determine whether a higher BMI contributes to anxiety in this population or whether anxiety itself leads to behaviors that promote weight gain, such as emotional eating, reduced physical activity, or metabolic dysregulation.

Endothelial responses to stress may link psychological stress to outcomes in coronary artery disease (Lima et al., 2019). Meta-analysis showed impaired endothelial function (measured by FMD) in those with clinical depression, with a 1.4% lower dilation response in general and a 3% reduction in those with clinical depression specifically. Those with depressive symptoms alone may not show this impairment, which may explain why our study found no significant FMD differences between participants with and without clinically relevant depression, as we did not distinguish between depressive symptoms and clinical depression (Waclawovsky et al., 2021).

Arterial stiffness is another parameter increasingly seen as a mechanism linking depression and anxiety to cardiovascular disease (CVD). Depressive and anxiety disorders are associated with a higher central augmentation index, indicating early wave reflection from arterial stiffness, and potentially promoting atherosclerosis progression (Seldenrijk et al., 2011). Studies show increased PWV in individuals with these conditions, with reductions in PWV observed among treatment responders (Yeragani et al., 2006; Schonthaler et al., 2023). In our study, we observed higher PWV in patients with clinically relevant anxiety but not in those with clinically relevant depression. A larger sample or confirmed depression diagnoses might reveal similar differences in the latter group.

Among blood markers, elevated fibrinogen levels have been linked to psychological distress, and notably, higher fibrinogen levels 1 month post-stroke are independently associated with emotional impairment (Wium-Andersen et al., 2013; Luan et al., 2020). Our findings support this, showing significant correlations between fibrinogen and HADS scores. Recent research also suggests a role for fibrinogen in neuroinflammation and mood disorders, with its interactions with astrocytes and neurotransmitters, such as serotonin, potentially impairing neural communication and contributing to depressive symptoms. It has been speculated that targeting fibrinogen pathways may hold promise as a therapeutic approach (Patel et al., 2024).

Research shows that elevated endocan levels may indicate endothelial dysfunction and be linked to CVD (Chen et al., 2021). Endocan, a proteoglycan secreted by activated endothelial cells, serves as a biomarker for endothelial dysfunction and inflammation. Its expression is modulated by pro-inflammatory cytokines such as TNF-α and IL-1β, which are also implicated in mood disorders (Chen et al., 2021; Sahan et al., 2023). While elevated endocan levels have been observed in psychiatric conditions like depressive disorders (Sahan et al., 2023), our study found significantly lower endocan concentrations in patients with clinically relevant anxiety, and a non-significant trend towards lower levels in those with depression. This inverse relationship may reflect a distinct inflammatory or vascular profile in post-MI patients during the early rehabilitation phase. Given endocan’s role in modulating endothelial permeability and leukocyte adhesion, its decreased levels could indicate a compensatory mechanism or a different trajectory of endothelial response in the context of acute cardiovascular events (Chen et al., 2021). These findings highlight the need for further research into the interplay between vascular biomarkers and psychological distress in cardiac populations.

BDNF, essential for neuronal development and plasticity, is also linked to CVD risk, with lower levels often associated with CVD (Bahls et al., 2019; Brigadski and Lessmann, 2020). Furthermore, BDNF is considered a potential biomarker for depression. While low BDNF levels are linked to post-stroke depression, its role in other CVDs remains underexplored. Our study did not show a significant decrease in clinically relevant depression but found a notable correlation between anxiety and both the total and anxiety subscale HADS scores. Although BDNF levels tend to be reduced in anxiety disorders, this trend is inconsistent. Research in this area is still in its early stages, and definitive conclusions about BDNF’s role in depression and anxiety are yet to be established (Suliman et al., 2013).

Our study has several limitations. First, the HADS threshold of 8 or above suggests possible symptoms, but does not provide a definitive diagnosis, as a full clinical evaluation by a mental health professional is required. While HADS helps to identify patients for further evaluation, it does not replace a full diagnostic assessment. Additionally, the HADS has lower sensitivity for detecting clinically relevant anxiety and depression, particularly in disease-specific studies, and it also does not assess somatic symptoms like fatigue and sleep disturbances potentially leading to missed cases (Bernstein et al., 2018; Moulton et al., 2019). The small sample size, single-center setting, and homogeneous population may limit generalizability. Although we excluded patients with previously diagnosed psychiatric disorders, undiagnosed or subclinical conditions could have influenced HADS scores and physiological parameters. The cross-sectional study design precludes any conclusions about causality or directionality between psychological distress and cardiovascular parameters. The absence of a control group further limits the ability to distinguish whether observed associations are specific to post-MI patients or reflect broader population trends. Self-reported measures such as the HADS are subject to reporting bias, particularly in a rehabilitation setting where psychological states may fluctuate. Residual confounding factors—including socioeconomic status, medication adherence, or lifestyle factors—may have influenced the observed associations and were not fully accounted for. Finally, while we identified differences in clinical parameters, causal relationships between these conditions and the parameters require further investigation.

## Conclusion

Screening for anxiety and depression in CR is essential given their impact on outcomes and adherence in CAD patients. Our findings reveal that patients with clinically relevant anxiety, compared to those without, tended to be younger, had higher BMI, greater arterial stiffness, elevated levels of fibrinogen, and reduced levels of endocan and BDNF. Meanwhile, those with clinically relevant depression showed higher systolic blood pressure and fibrinogen levels. Additionally, age was inversely correlated with HADS scores for both depression and anxiety. Addressing mental health in CR could offer notable benefits, potentially leading to improvements in cardiovascular health markers. Future studies should explore the long-term impact of integrating psychological screening and targeted interventions into cardiac rehabilitation, with particular focus on how these strategies influence both mental health trajectories and cardiovascular outcomes over time.

## Data Availability

The raw data supporting the conclusions of this article will be made available by the authors, without undue reservation.

## Abbreviations

BMI, Body mass index; BDNF, Brain-derived neurotrophic factor; CAD, Coronary artery disease; CR, Cardiac rehabilitation; CVD, Cardiovascular disease; FMD, Flow-mediated dilation; HADS, Hospital Anxiety and Depression Scale; MI, Myocardial infarction; PWV, Pulse wave velocity; BP, Blood pressure; BPM, Beats per minute.
